# Effect of sex on the gut microbiota characteristics of passerine migratory birds

**DOI:** 10.3389/fmicb.2022.917373

**Published:** 2022-09-02

**Authors:** Rongfei Yan, Meixia Lu, Lishi Zhang, Jiyuan Yao, Shi Li, Yunlei Jiang

**Affiliations:** ^1^College of Animal Science and Technology, College of Veterinary Medicine, Jilin Agricultural University, Changchun, China; ^2^School of Life Sciences, Jilin Agricultural University, Changchun, China

**Keywords:** migratory birds, passerine, stopover sites, sex, gut microbiota

## Abstract

The gut microbiota, considered the “invisible organ” in the host animal, has been extensively studied recently. However, knowledge about the gut microbiota characteristics of passerine migratory birds during migration is limited. This study investigated the gut microbiota characteristics of three dominant migratory bird species (namely orange-flanked bluetail *Tarsiger cyanurus*, yellow-throated bunting *Emberiza elegans*, and black-faced bunting *Emberiza spodocephala*) in the same niche during spring migration and whether they were bird sex-specific. The compositions of gut microbiota species in these three migratory bird species and their male and female individuals were found to be similar. The main bacterial phyla were Proteobacteria, Firmicutes, Actinobacteria, and Bacteroidetes, and the main genera were *Lactobacillus*, *Acinetobacter*, *Rickettsiella,* and *Mycobacterium*; however, their relative abundance was different. Moreover, some potential pathogens and beneficial bacteria were found in all the three bird species. Alpha diversity analysis showed that in *T. cyanuru*s, the richness and diversity of the gut microbiota were higher in male individuals than in female individuals, while the opposite was true for *E. elegans* and *E. spodocephala*. The alpha diversity analysis showed significant differences between male and female individuals of *E. elegans* (*p* < 0.05). The beta diversity analysis also revealed that the gut microbial community structure differed significantly between the male and female individuals of the three migratory bird species.

## Introduction

The gut microbiota in vertebrates may affect their metabolism, development, and physiological processes, including health and behavior (Bäckhed et al., 2005; Heijtz et al., 2011; Tremaroli and Bäckhed, 2012). The gut microbial community of birds has some unique and distinctive features compared to that of mammals, fish, and insects (Hird et al., 2014), which are possibly related to their diet, physiological characteristics, sex, and reproductive patterns (Kohl, 2012). The gut microbiota of wild birds has recently received widespread attention for being the source of zoonotic pathogens that cause many human and animal diseases (Tsiodras et al., 2008).

With diverse species and life history characteristics, birds can have different migratory behavior, flying capacity, diet, mating system, lifespan, and physiology, all of which may influence the gut microbiome (Grond et al., 2018). According to Hologenome theory, organisms and their microbiota communities co-evolve and these communities can improve host adaptive parameters such as survival, phenotypic plasticity, and reproductive performance in response to environmental stresses such as migration (Zilber-Rosenberg and Rosenberg, 2008; Grond et al., 2018). In birds, migration can significantly impact the gut microbiota composition due to a change in intrinsic physiology affecting the plasticity of gut morphology. For example, in migratory seabirds, the gastrointestinal tract mass (length) can reduce up to 30% before long-distance migration (Battley et al., 2000). This significantly impacts the bird’s physiological function and food intake rates, thereby altering the physical habitat of gut microorganisms. In addition, the lack of nutrient intake during flight may make the microbiota community monotonous. Accordingly, many migratory birds often have one or more migration stopover sites (Schmaljohann et al., 2022) that are often rich in food. At a stopover site, the interaction between birds can promote the transfer of microorganisms through close contact and involuntary co-feeding (Grond et al., 2014; Ryu et al., 2014). At the halfway stop site, the microbiota already present in the gastrointestinal tract of migratory birds may compete vigorously with incoming food-associated microbiota. Alternatively, the small residence time of the migratory bird at the stopover site may not allow the rapid settling of food intake-related bacteria.

Migration is a costly behavior as it consumes a lot of energy. However, in birds, it allows access to abundant seasonal resources (Gauthreaux, 1980). Seasonal bird migration usually involves a sequence of movements with stopovers to replenish energy and rest. Therefore, stopovers are crucial for the successful migration of animals (Hegemann et al., 2018). Theoretical models suggest that birds spend 90% of their total migration time and 67% of their energy during migratory stops (Hedenström and Alerstam, 1997). Migration can reduce the risk of infection as birds can escape from pathogens in time and space through migration, which is known as the “migratory escape” (Altizer et al., 2011; Poulin et al., 2012; Shaw and Binning, 2016). For many species such as mammals (Mysterud et al., 2016), insects (Bradley and Altizer, 2005), fish (Sjöberg et al., 2009), and birds (Risely et al., 2018), if an animal escapes from a habitat where the pathogen has accumulated, or if the arduous journey kills the infected host, migration can reduce the risk of infection (Satterfield et al., 2018). Additionally, if complex environmental changes during migration are unsuitable for pathogens (Shaw and Binning, 2016), the infected hosts may die during migration and their death reduces the risk of infection in successfully migrating individuals (Johns and Shaw, 2015). Nevertheless, some researchers believe that migration may increase the host’s susceptibility to pathogens (Figuerola and Green, 2000) as birds will regulate and redistribute immune function during migration (Buehler et al., 2010; Eikenaar and Hegemann, 2016). However, the physiological pressure of migration can weaken the defense capability of the host, increasing the risk of infection (Buehler and Piersma, 2008). Moreover, migratory birds may encounter more pathogen infestations at breeding and wintering sites and along migration routes (Bauer and Hoye, 2014; Shaw and Binning, 2016). For example, the migration of sandhill crane (*Grus canadensis*) promoted *Enterococcus* spp., *Escherichia coli,* and *Campylobacter* spp. in the river water and at its stopover sites (Lu et al., 2013).

A series of intrinsic (genetics, age, sex, and health) and extrinsic (diet, social activities, and environmental microorganisms) factors and their interactions can influence the composition and structure of gut microbiota (Benson et al., 2010; Bevins and Salzman, 2011; Bolnick et al., 2014; Grond et al., 2018). The impact of sex, an internal factor, on gut microbiota is often ignored (Kim et al., 2020) and fiercely debated. Animal sex can influence the gut microbiota composition through sex-specific hormone–microbial interaction and immune response (Org et al., 2016). In mice, even with the same diet and feeding environment, the gut microbiota composition differed significantly between male and female individuals (Org et al., 2016). By contrast, some studies have suggested no effect of animal sex on gut microbiota (Lay et al., 2005; Kreisinger et al., 2015). External environmental factors, such as feeding location and food, are considered the major factors affecting the gut microbiota of passerines (Hird et al., 2014). The gut microbiota of songbirds showed more similarities within and between species during stopovers, suggesting a crucial role of the local diet and/or environment in gut microbiota changes (Lewis et al., 2017). Notably, birds lack initial mechanical digestion and may be more dependent on the gut microbial community for digestive functions (Grond et al., 2018).

Male and female animals have different reproductive physiology and behavior, which may affect their gut microbiota characteristics. Among the eight most common bacterial genera, three genera (*Enterococcus*, *Rothia,* and *Streptococcus*) differed significantly between male and female individuals in *Colinus virginianus* (Su et al., 2014). Sex hormones are known to interact with human gut microbiota (Neuman et al., 2015), but the same is unclear in birds. Studies investigating avian sex hormones have concentrated on the immunosuppressive effects of testosterone (Alonso-Alvarez et al., 2009). Escallón et al. (2016) concluded that testosterone levels positively correlate with cloacal microbiota diversity and *Chlamydia* spp. abundance, producing a potential immunosuppressive effect (Escallón et al., 2016). Alternatively, higher testosterone levels may increase the mating sessions with different mates, facilitating bacterial transmission through sex. Overall, the behavior of animals can significantly affect their microbiota communities and vice versa (Ezenwa et al., 2012).

The bird gut microbiota is mainly composed of Firmicutes, Proteobacteria, Bacteroidetes, and Actinobacteria (Grond et al., 2018). Several poultry studies have shown that Firmicutes abundance is positively correlated with animal weight gain and immune function, but the same is not known in wild birds (Grond et al., 2018). The proportion of Proteobacteria is higher in birds than in mammals, especially Proteobacteria can account for up to 45% in wild birds (Grond et al., 2018). Although Actinobacteria are the fourth most abundant microbial phylum in the gastrointestinal tract of wild birds, their effect on bird physiology is largely unknown (Grond et al., 2018). Many gut commensal microorganisms, such as Bifidobacteriaceae and Lactobacillaceae, are considered probiotics with essential roles in nutrition, growth, and prevention of infection (Bäckhed et al., 2005; Quigley, 2010; Olnood et al., 2015).

The gut microbiome characteristics have been intensively studied in humans (Zhang et al., 2019; Wang et al., 2022), and small-scale studies have been conducted in poultry too (Borda-Molina et al., 2018; Lee et al., 2019). However, the characteristics of gut microbiota in passerine migratory birds during migration remain unclear. In this study, at the stopover site of migratory birds in Heilongjiang Province, China, we selected three dominant passerine bird species, namely the orange-flanked bluetail (*Tarsiger cyanurus*) and two migratory birds of the same genus, the yellow-throated bunting (*Emberiza elegans*) and black-faced bunting (*Emberiza spodocephala*) as the research objects. Two subspecies of *T. cyanurus* can be found in China: *T. c. cyanurus* and *T. c. rufilatus*. We here studied *T. c. cyanurus*, which breeds in Siberia and overwinters in the south of Yangtze River. During the migration period, the *T. cyanurus* population peaked in mid-to-late April and stayed for (3.4 ± 3.4) days (Wang et al., 2006; Luo et al., 2014), and the average migration time was 26.45 ± 3.46 (X ± SD, *N* = 20). *E. elegans* in the study area is a northeastern subspecies (*E. e. ticehursti*) that overwinters in North, Central, and Southwest China and breeds in the Russian Far East and Northeast (Choi et al., 2019). The population of male birds peaked in late March and that of female birds peaked in mid-April, with an average migration time of 33.6 ± 4.54 (X ± SD, *N* = 20). *E. spodocephala* has three subspecies, and the research object was a nominate subspecies. These birds are mainly distributed in Eurasia; breed in Siberia, northeastern China, and Korea; and overwinters in the south (Choi et al., 2020). The *E. spodocephala* population peaked in mid-to-late April, with an average migration time of 29.95 ± 3.34 (X ± SD, *N* = 20). The 16S rRNA gene fragments of microorganisms in the field-collected samples of feces were amplified and sequenced through high-throughput sequencing technology to analyze the characteristics of the gut microbiota of migratory birds in the same ecological niche, and the possible effect of sex factors on gut microorganisms in these birds.

## Materials and methods

### Sample collection

The fecal samples were collected at the Maoer Mountain Bird Ringing Center (45°24′13.3″N, 127°39′39.7″E) of Northeast Forestry University during the spring migration of migratory birds in April 2021. The migratory birds were captured in fog nets, and after ringing, sterile collection containers were placed at the bird’s anus to directly collect the feces by gently pressing the bird’s belly. After labeling the samples, they were immediately placed into liquid nitrogen for storage. After being transported to the laboratory, the fecal samples were stored at −80°C for later use. In total, 120 samples (40 samples of each *T. cyanurus*, *E. elegans*, and *E. spodocephala,* including 20 from males and 20 from females) were harvested.

### DNA extraction, amplification, and genome sequencing

The DNeasy PowerSoil Kit (Mo Bio) was used to extract microbial DNA from the fecal samples following the manufacturer’s instructions (Wei et al., 2020; Meng et al., 2021; Obrochta et al., 2022). The DNA concentration was estimated using a fluorescence spectrophotometer (Quant-iT PicoGreen dsDNA Assay Kit, dsDNA), and the quality of the product was analyzed through 1% agarose gel electrophoresis. The DNA samples were uniformly diluted to 20 ng/μl for subsequent analysis. The v3–v4 region of the bacterial 16S rRNA gene was amplified through PCR by using forward 338F (5′-ACTCCTACGGGAGGCAGCA-3′) and reverse 806R (5′-GGACTACHVGGGTWTCTAAT-3′) primers (Ding et al., 2020; Feng et al., 2021; Meng et al., 2021); the PCR system and conditions are described in Supplementary material. The PCR amplification products were recovered and purified using the AxyPrep DNA Gel Extraction Kit (Axygen, AP-GX-500), and each sample was quantified with the Quant-iT PicoGreen dsDNA Assay Kit (Invitrogen, P7589) by using a BioTek Flx800 microplate reader. The high-quality DNA samples were used to construct libraries following the standard Illumina DNA library preparation experimental procedures, and sequencing was performed on the Illumina NovaSeq PE250 (2 × 125 bp; San Diego, California, United States) platform. All data are stored in the Sequence Read Archive database of NCBI with ID PRJNA772042.

### Sequence data processing

The gene sequences obtained from sequencing on the Illumina NovaSeq PE250 platform were analyzed using QlIME2 2019.4 (Callahan et al., 2017; Bolyen et al., 2019; Tipton et al., 2022) software. Raw sequence data were demultiplexed using the demux plugin. Primers were cut using cutadapt 1.16 (Martin, 2011). Next, the DADA2 plugin (Callahan et al., 2016) was used for quality filtering, denoising, merging, and removal of chimera. After dereplication, amplicon sequence variants (ASVs) were obtained. According to the Greengenes database (DeSantis et al., 2006), using the classify-sklearn algorithm of QIIME2 2019.4 (Bokulich et al., 2018) and default parameters in the pre-trained Naive Bayes classifier, species annotation was performed on the characteristic sequence of each ASV. In QIIME2 2019.4, the obtained non-singleton ASVs were aligned with mafft (Katoh et al., 2002) and then used to construct a phylogenetic tree with FastTree2 (Price et al., 2009). Using the Rarefaction method, the generated ASV abundance table was subsampled (Heck et al., 1975; Kemp and Aller, 2004). Next, in QIIME2 2019.4, we used the Qiime feature-table rarefy function to set the subsample depth to 95% of the minimum sample sequence size to obtain the ASV subsample table.

### Statistical analysis

According to the ASV subsample table, using the self-compiled Perl script and the results of taxonomic annotation of the sequences, the number of taxa in the respective sample at each taxonomic level was calculated. In the QIIME2 2019.4 program, the feature table after the removal of singletons is statistically performed, and the “qiime taxa barplot” command is invoked. In this article, the composition distribution of 10 species with the highest relative abundance at the taxonomic level of phylum and genus is shown, while the relative abundance of the remaining species was combined and classified as Others. The species composition of the sample is shown accordingly in a column chart. With the genus as the highest taxonomic level, the top 100 ASVs in abundance were taken and a circle packing chart was used to present the taxonomic composition of the microbial community, showing the proportion of different taxonomic units in different groups at the same time (Carrión et al., 2019). Using the ggplot2 package (Wickham et al., 2016) in R v3.2.0., a microbial taxonomic rank tree was drawn and the grouped abundance data for respective ASVs were added to the graph as pie charts.

Richness and diversity are characterized by Chao (1984) and Shannon (1948) indices, respectively. In QIIME2 2019.4, using the unsubsample ASV table, the “qiime diversity alpha-rarefaction” command was invoked and the minimum subsample depth was set to 10, 95% of the sequence volume of the sample as the lowest sequencing depth to calculate the alpha diversity index. Data were plotted as boxplots using the ggplot2 package (Wickham et al., 2016) in R v3.2.0., and the significance of differences was verified using the Kruskal–Wallis rank-sum test and Dunn’s test as *post-hoc* tests. To explore whether the size of the sample alpha diversity index was related to the subsample depth of the ASV table, a rarefaction curve was drawn to predict the total number of species and the relative abundance of respective species (Heck et al., 1975; Kemp and Aller, 2004). The “qiime diversity alpha-rarefaction” command in QIIME2 2019.4 was used to generate the alpha-rarefaction.qzv file, which was visualized at https://view.qiime2.org/.

The beta diversity analysis is conducted for comparing diversity between different habitats, that is, the differences between different samples. Principal coordinates analysis (PCoA) is one of the most classic non-constrained ordination (Classical Multidimensional Scaling, cMDScale) analysis methods (Ramette, 2007) that can be used to reduce the dimensionality of multidimensional microbial data. PCoA was employed to find the main trends by distributing samples in a continuous ordination axis. In QIIME2 2019.4, the unweighted UniFrac distance matrix was calculated by invoking the “qiime diversity core-metrics-phylogenetic” command based on the tree file by using the ASV subsample table (Lozupone and Knight, 2005; Lozupone et al., 2007) to perform PCoA analysis and produce output QZV files. Meanwhile, the PCoA coordinates of the output sample points were analyzed through PCoA by using the ape package in R v3.2.0. and plotted as a two-dimensional scatter plot. If the purpose of ordination is to find continuity in the data (i.e., showing the main trends of the data through a continuous ordination axis), then the purpose of cluster analysis is to find discontinuity in the data. Hierarchical clustering is often used in beta diversity clustering analysis to display the similarity between samples in the form of a hierarchical tree. The quality of the clustering effect is determined by measuring the branch length of the clustering tree. We used the unweighted pair-group method with arithmetic means to compute the unweighted UniFrac distance matrix for clustering analysis (Li and Xu, 2010). Through the unweighted UniFrac distance matrix, permutational multivariate analysis of variance (PERMANOVA) was used to analyze the differences in the microbial community structure between male and female birds (McArdle and Anderson, 2001); the differences between groups were analyzed using the scikit-bio package in Python v2.7.15. The number of permutation tests was 999.

Having explored differences in microbial community composition (i.e., beta diversity), we next examined the species primarily responsible for these differences. We used Venn diagrams (VennDiagram package in R v3.2.0) for microbial community analysis to identify the common and unique species among different groups. The abundance data of ASV in all samples were used to calculate the relationship between male and female individuals (Zaura et al., 2009). The linear discriminant analysis (LDA) effect size (LEfSe) analysis is a differential analysis method that combines the nonparametric Kruskal–Wallis and Wilcoxon rank-sum tests with the LEfSe (Segata et al., 2011). The LEfSe analysis can directly and simultaneously perform differential analysis on all taxonomic levels, identifying robust differential species between groups, namely biomarkers. We used a species taxonomic cladogram to show the taxonomic hierarchical distribution of marker species in each group of samples. The LEfSe package in Python v2.7.15 (Segata et al., 2011) with the Kruskal–Wallis rank-sum test (*p* < 0.05) was used to detect all characteristic species and species with significant differences between groups by detecting the difference in species abundance. Next, the Wilcoxon rank-sum test (*p* < 0.05) was used to detect the significance and consistency of the obtained data, and lastly, the LDA (default LDA threshold) was used to obtain the final differential ASV (i.e., biomarkers; Segata et al., 2011).

Results were considered significant at *p* < 0.05.

## Results

### Species composition and relative abundance of gut microbiota communities in migratory bird species

**Figure 1:**
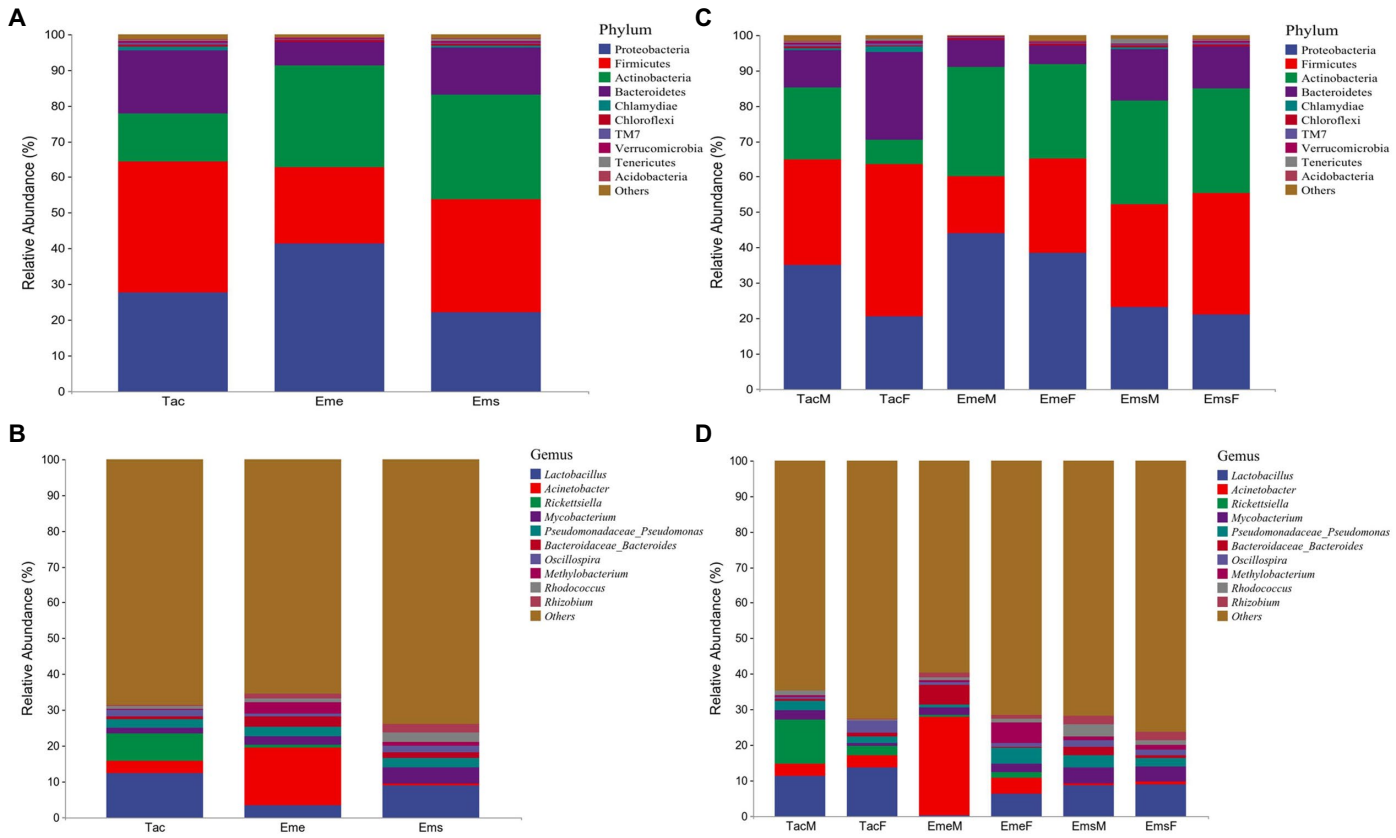
Relative abundance of major taxa in bird’s gut microbiota. The abscissa in each figure represents the respective group, and the ordinate shows the relative abundance of respective taxa at the classification level. **(A)** Relative abundances at the phylum level for the three migratory bird species; **(B)** Relative abundances at the phylum level between male and female individuals of the three bird species; **(C)** Relative abundances at the genus level for the three migratory bird species, and **(D)** Relative abundances at the genus level between male and female individuals of the three bird species. Tac, *Tarsiger cyanurus*; Eme, *Emberiza elegans*; Ems, *Emberiza spodocephala*; TacM, *T. cyanurus* male; TacF, *T. cyanurus* female; EmeM, *E. elegans* male; EmeF, *E. elegans* female; EmsM, *E. spodocephala* male; EmsF, *E. spodocephala* female.

In total, after quality processing, 13,207,177 clean sequencing reads were obtained from 120 samples from the three bird species with an average of 110,059 reads per sample. Cumulative histograms were used to present gut microbial species composition at phylum and genus levels. The dominant phyla in the three migratory bird species were Proteobacteria, Firmicutes, Actinobacteria, and Bacteroidetes, among which Chlamydiae, Chloroflexi, TM7, and Verrucomicrobia were in small amounts, a set of unclassified sequences accounted for the remainder (Figure 1A). The taxonomic hierarchy tree diagram depicts the percentages of members in the three migratory bird species at the phylum, order, family, and genus levels (Supplementary Figures 1A-a–c). The dominant phylum in *T. cyanurus* was Firmicutes (36.56%), Proteobacteria (41.32%) in *E. elegans*, and Firmicutes (31.74%) in *E. spodocephala*, while the remaining bacteria and unclassified sequences accounted for a small amount (Figure 1A). The dominant genera in the three migratory bird species were also the same (Figure 1B) including *Lactobacillus*, *Acinetobacter*, *Rickettsiella*, *Mycobacterium,* and *Pseudomonadaceae_Pseudomonas*, among which, *Bacteroidaceae_Bacteroides*, *Oscillospira*, *Methylobacterium*, and *Rhodococcus* were in small amounts. The dominant genus was *Lactobacillus* (12.39%) in *T. cyanurus, Acinetobacter* (16.12%) in *E. elegans*, and *Lactobacillus* (8.76%) in *E. spodocephala*, while the remaining sequences accounted for other bacteria and unclassified sequences (Figure 1B).

Concerning the species composition difference at the sex level, the main phylum of gut microbiota was the same in both males and females in *T. cyanurus*, *E. elegans*, and *E. spodocephala* but differed in relative abundance (Figure 1C). A taxonomic hierarchy tree diagram depicting the percentage of respective phylum, order, family, and genus in the male and female birds of the three bird species is shown in Supplementary Figures 1B-a–c. In *T. cyanurus* (Figure 1C), Firmicutes were most abundant in female birds (43.16%), while Proteobacteria were most abundant in male birds (35.00%). Notably, Tenericutes and Spirochaetes were detected only in female birds. In *E. elegans* (Figure 1C), Proteobacteria were relatively abundant in both male (44.08%) and female (38.57%) birds, whereas Spirochaetes and Fusobacteria were detected only in female birds. In *E. spodocephala* (Figure 1C), Firmicutes were dominant in both male (29.17%) and female (34.32%) birds, whereas Chlamydiae were detected only in male birds, and Acidobacteria were detected only in female birds. As shown in Figure 1D, the major genera were similar in both male and female birds of *T. cyanurus*, *E. elegans,* and *E. spodocephala* but differed in relative abundance. In *T. cyanurus*, *Lactobacillus* and *Rickettsiella* were the dominant genera in female (13.53%) and male (12.37%) birds, respectively. *Acinetobacter* and *Lactobacillus* were relatively abundant in male (27.83%) and female (6.30%) birds of *E. elegans*, respectively; and in *E. spodocephala*, *Lactobacillus* was quite relatively abundant in both male (8.57%) and female (8.95%) birds.

### Richness and diversity of gut microbial communities in migratory birds

**Figure 2:**
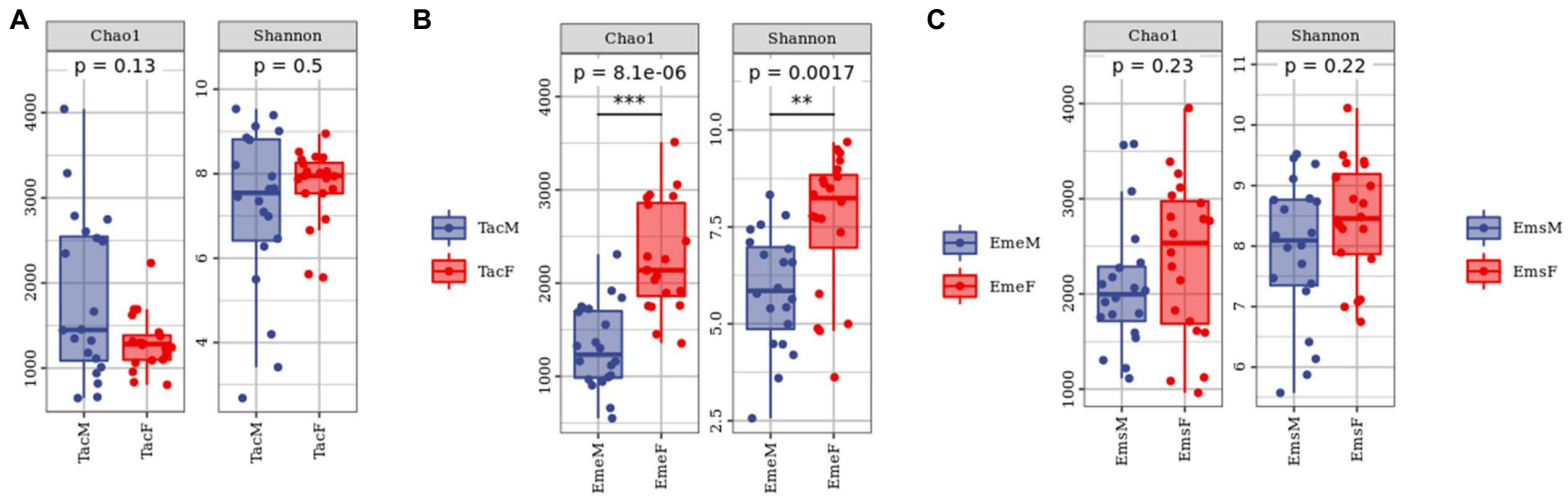
Alpha diversity analysis of gut microbiota in migratory birds. Each dot denotes a sample. Each panel corresponds to an alpha diversity index indicated by the gray area at its top. In each panel, the abscissa is the grouping, and the ordinate is the value of the corresponding alpha diversity index. The numbers under the Diversity Index labels are *p*-values of the Kruskal–Wallis test. Alpha diversity analysis between male and female individuals of **(A)** *Tarsiger cyanurus*, **(B)** *Emberiza elegans,* and **(C)** *Emberiza spodocephala*. The abbreviations in this figure are expanded in the legend of Figure 1. The numbers under the Diversity Index labels are *p*-values of the Kruskal -Wallis test. **significant difference, ***extremely significant difference.

The sparsity curves of male and female individuals were calculated using the Chao1and Shannon indices. The analysis showed that sequencing data captured most of the microbiota diversity in the sample (Supplementary Figure 2). To examine the gut microbiota alpha diversity among male and female individuals of the three migratory bird species (Figure 2; Supplementary Table 1), we used Chao1and Shannon indices for characterization. All three bird species exhibited high alpha diversity. Gut microbiota alpha diversity differed significantly between the male and female individuals of the same species. The richness and diversity of gut microbial communities were higher in male birds than in female birds in *T. cyanurus* (Figure 2A); however, in *E. elegans* (Figure 2B) and *E. spodocephala* (Figure 2C), it was the opposite. Alpha diversity indices differed significantly between female and male birds in *E. elegans*.

### Ordination and cluster analysis of microbial communities

**Figure 3:**
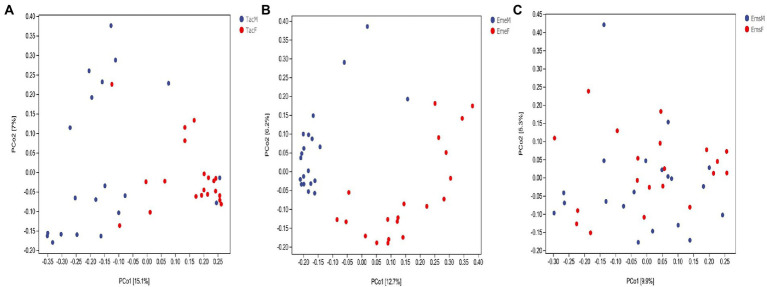
PCoA analysis of the gut microbiota of migratory birds. Each point denotes a sample. The percentages in brackets on the axes represent the proportion of the sample difference data (distance matrix) explaining the corresponding axes. The comparisons between male and female individuals of **(A)** *Tarsiger cyanurus*, **(B)** *Emberiza elegans*, and **(C)** *Emberiza spodocephala* are shown. The abbreviations in this figure are expanded in the legend of Figure 1.

Ordination analysis of gut microbes in male and female (Figure 3) birds was performed using PCoA based on the unweighted UniFrac distance algorithm. *T. cyanurus* (Figure 3A) and *E. elegans* (Figure 3B) showed distinct clustering between the male and female individuals, whereas *E. spodocephala* (Figure 3C) did not. Average clustering based on the unweighted UniFrac algorithm at the genus classification level identified the clear clustering of male and female individuals of *T. cyanurus* (Supplementary Figure 3A) and *E. elegans* (Supplementary Figure 3B), whereas the clustering of male and female individuals of *E. spodocephala* (Supplementary Figure 3C) remained chaotic. The results of the PERMANOVA test based on the unweighted UniFrac distance algorithm showed that the differences in the gut microbial community structure between the male and female birds of *T. cyanurus* and *E. elegans* were significant (*p* = 0.001), and those between male and female birds of *E. spodocephala* were statistically significant (*p* = 0.016; Supplementary Table 2). Thus, beta diversity analysis revealed that the gut microbial community structure differed significantly between the male and female birds in all three migratory bird species.

### LEfSe-based differential analysis of gut microbiota

To explore the shared and unique gut microbiota species between the male and female individuals of the three migratory bird species, a Venn diagram (Supplementary Figure 4) was constructed on the basis of ASV abundance data. In total, 3,619, 3,205, and 5,536 sequences are common between the male and female individuals of *T. cyanurus* (Supplementary Figure 4A), *E. elegans* (Supplementary Figure 4B), and *E. spodocephala* (Supplementary Figure 4C), respectively.

**Figure 4:**
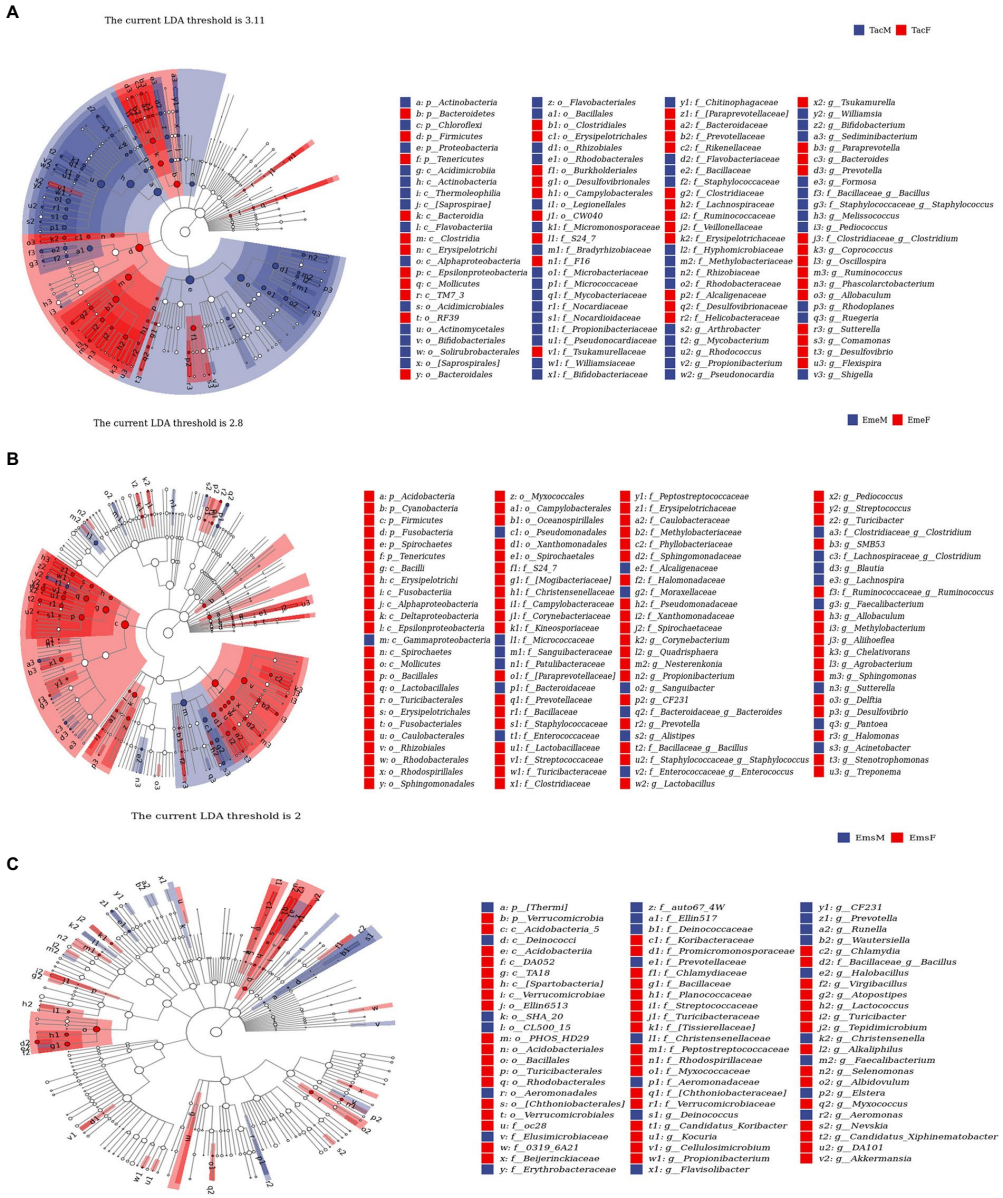
LEfSe species difference analysis-species taxonomy cladogram showing the taxonomic-level distribution of marker species between male and female individuals of **(A)** *Tarsiger cyanurus*, **(B)** *Emberiza elegans*, and **(C)** *Emberiza spodocephala*. The three migratory bird species have their own optimal thresholds (above the cladogram). Circles radiating from inside to outside represent the taxonomic hierarchy of the major taxonomic units from the phylum to genus in respective communities. p_:phylum, c_:order, o_:order, f_:family, g_:genus. The size of the nodes in the cladogram corresponds to the average relative abundance of ASV at the respective taxonomic level. The diameter of the node is proportional to the relative abundance. The hollow nodes represent species showing no significant differences, and the solid circles with different colors represent the relative abundances of significantly different species. Letters or letters/numbers at nodes in cladogram: labels for all different ASVs from the phylum to genus. Letter–number combinations were assigned per Figure part and do therefore not correspond to each other between the different Figure parts **(A–C)**. Species with taxonomic names in brackets have no clear taxonomic understanding yet but exist in the database, that is, suspected taxonomy of the species (further validation is required). We kept the original Greengenes assignments, even if genera were assigned to wrong classes. We ensured that this did not change our results or conclusions. The abbreviations in this figure are expanded in the legend of Figure 1.

LEfSe (Segata et al., 2011) analysis was used to identify microbial communities with significant differences in relative abundance between the male and female individuals of the three migratory bird species (Figure 4). We described the data at the phylum and genus levels. For example, at the phylum level, in *T. cyanurus* (Figure 4A), Actinobacteria and Proteobacteria showed a significant difference, with the highest relative abundance in male birds; Bacteroidetes and Firmicutes had the highest relative abundance in female birds. For example, at the genus level, a significant difference was observed for *Mycobacterium*, with the highest relative abundance in male birds, while *Oscillospira*, *Ruminococcus,* and *Allobaculum* had the highest relative abundance in female birds. For example, in *E. elegans* (Figure 4B), the phylum Firmicutes showed the highest relative abundance in female birds and the genus *Acinetobacter* showed the highest relative abundance in male birds, while *Lactobacillus* and *Methylobacterium* were higher in female birds. Likewise, for example, in *E. spodocephala* (Figure 4C), the phylum Verrucomicrobia showed the highest relative abundance in female birds. The genera *Prevotella*, *CF231*, and *Faecalibacterium* showed significant differences, with the highest relative abundance in male birds, while *Bacillus* and *Turicibacter* showed the highest abundance in female birds.

## Discussion

Many recent studies have investigated on the gut microbiota of migratory waterbirds (Wang et al., 2016, 2019; Cao et al., 2020), but only a few have focused on small Passerine migratory birds. This study examined the gut microbiota structure and composition of *T. cyanurus*, *E. elegans*, and *E. spodocephala* in the same niche, as well as the effect of sex differences in birds on gut microbiota.

### Gut microbial community characteristics of migratory bird species during migration

With long-term co-evolution of species, the host and its gut microbiota have established a relatively stable symbiotic system. Our results showed that the main dominant bacterial groups in the three migratory bird species are the same, mainly Proteobacteria, Firmicutes, Actinobacteria, and Bacteroidetes, which account for >96%; however, the relative abundance of dominant microbiota was different in each species. This is consistent with the results of a meta-analysis of bird gut microbiota conducted by Waite and Taylor (2014). Studies have shown that gut microbes play a vital role in avian host nutrition, immune function, and toxin processing (Kohl, 2012).

Firmicutes play a major role in the metabolism, digestion, and absorption of proteins and other nutrients (Berry, 2016). Short-chain fatty acids produced by Firmicutes can be directly absorbed by the gut wall to provide energy to the host, and the abundance of Firmicutes is associated with weight gain in humans, chickens, and rodents. Firmicutes abundance is positively related to mass gain and immune function in both mammals and chickens, but the role of Firmicutes in wild birds is unclear (Grond et al., 2018).The relatively high abundance of Firmicutes in insectivorous *T. cyanurus* and omnivorous *E. spodocephala* may help these birds with the required energy during migration. Proteobacteria were dominant in all the three migratory bird species but were significantly enriched in *E. elegans*. The increased abundance of Proteobacteria is considered a marker of biological disorder or potential disease (Shin et al., 2015), and Proteobacteria are dominant in malnourished children (Monira et al., 2011). Bacteroidetes degrade carbohydrates, breakdown polysaccharides that cannot be digested by the host to obtain energy, and interact with the immune system to inhibit the colonization of potential pathogens, and their metabolites (such as acetic acid, propionic acid, and succinic acid) contribute to killing of pathogens (Nkosi et al., 2022). Our research found that Bacteroidetes were significantly enriched in all the three migratory bird species. Actinobacteria play a key role in maintaining the stability of the gut’s internal environment (Binda et al., 2018) as well as in the breakdown of organic materials such as cellulose and chitin. Some symbiotic Actinobacteria species, namely probiotics, can control bacterial diseases in the host (Anandan et al., 2016). Actinobacteria were significantly enriched in *E. spodocephala* and *E. elegans*. The beneficial bacterium *Lactobacillus* was abundant in all the three migratory bird species, which can ferment lactose to produce lactic acid that has antibacterial, antiviral, and immunomodulatory effects (Tachedjian et al., 2017). In general, *Lactobacillus* accounts for 10% of the human gut microbiota (Kontula et al., 2000). It is also used as a feed additive to improve the immune safety of the host (Merk et al., 2005). Lactobacilli were also found in the gut microbiota of Swainson’s thrush (*Catharus ustulatus*) and gray catbird (*Dumetella carolinensis*) during spring and autumn migration (Lewis et al., 2016). Similarly, among the 11 dominant genera in the gut microbiota of the wintering *Grus nigricollis*, *Lactobacillus* was present in the highest proportion (Wang et al., 2020). In addition, we discovered some pathogenic bacteria, such as *Acinetobacter* spp. and *Rickettsia*. *Acinetobacter* spp. are gram-negative non-fermentative bacteria that are prevalent in hospitals as opportunistic pathogens (Bergogne-Bérézin, 1994). Ticks play an important role in the transmission of *Rickettsia* disease (Giudice et al., 2014). These pathogens may be potential sources of infections that cause zoonotic diseases.

### Effect of sex factors

Studies in mice, chickens, and pigs show that host sex can influence gut microbiota colonization (Pajarillo et al., 2014; Org et al., 2016; Zhou et al., 2016). Bird gut microbiomes also exhibit substantial intraspecific variation (Song et al., 2020). This study found a higher abundance and diversity of gut microbiota in male *T. cyanurus*, while *E. elegans* and *E. spodocephala* showed the contrary trend. The present study findings of *E. elegans* and *E. spodocephala* are consistent with those of Kim’s review (Kim et al., 2020).

The beta diversity analysis revealed sex differences in the gut microbial community structure of the three migratory bird species. However, the effect of sex on the host gut microbial community structure is controversial. Consistent with our findings, Yong et al. also suggested that sex can affect the gut microbial community structure (Kim et al., 2020). Differences were found in the gut microbial community composition and individual ASV abundance between female and male birds of thick-billed murres (*Uria lomvia*; Góngora et al., 2021). On the contrary, some studies have shown that sex does not affect the structure of the intestinal microbial community. For example, sex differences in the migratory barn swallow (*Hirundo rustica*) have no effect on the cloacal microbial community structure (Kreisinger et al., 2015); likewise, no difference was observed in the colonic microbiota of two sexes in a community of 91 Nordic people (Lay et al., 2005) and wild adult gorillas (Pafčo et al., 2019). Venn diagrams showed the core gut microbiota between male and female birds. Astudillo-Garcia et al. suggested that the core microbiota may play a critical role in responding to environmental changes, and the reservoir may stabilize the host’s microbiome (Astudillo-García et al., 2017). LEfSe analysis of differences in the gut microbiota showed that male and female birds have distinct differences in the gut microbial community at both phylum and genus levels. Whether the differences in the gut microbial community structure between the male and female birds of the three migratory bird species are related to sex or diet requires further research. Hird et al. explored the factors influencing the gut microbial communities of 59 Neotropical bird species in terms of 18 categorical variables including host species, diet, environment, and geographical location. They found that the host species was the most dominant determinant of the microbial community composition, followed by ecological factors (i.e., diet and habitat; Hird et al., 2015).

The effects of sex differences on host gut microbial communities is naturally associated with the role of sex hormones (Kim et al., 2020). The interaction of sex hormones with host gut microbes has been extensively studied in humans and mice but such studies are rare in birds. A 20-year study of hormonal effects in the golden-collared manakins *Manacus vitellinus* found significant differences in male-typical behavior between wild and captive male and female manakins (Schlinger and Chiver, 2021). In humans, bilateral ovariectomy was found to be associated with an increased abundance of *Clostridium bolteae* (Sinha et al., 2019). After gonadectomy, the composition of gut microbial communities significantly changed in testosterone-treated vs. untreated men (Org et al., 2016). In all, an interaction seems to occur between gut microbiota and sex hormones, but further studies are required to confirm this.

## Conclusion

This study partially fills the gap in the gut microbial community characteristics of three migratory bird species on the East Asia-Siberia route during migration. The study highlights that the influence of sex on the host gut microbiota cannot be ignored. Importantly, all the three bird species were found to carry different levels of potentially pathogenic bacteria. Migratory birds may be infected by exposure to pathogens from the environment and/or other birds during migration, and then may act as a reservoir of pathogens that can be transmitted to other birds or humans (Cao et al., 2020; Smith et al., 2020; Turjeman et al., 2020). Therefore, collecting samples during migratory stopovers can detect the health status of migratory birds and identify potential pathogens. Future studies should comprehensively analyze the characteristics of gut microbial communities of migratory birds at spatial and temporal levels throughout the migration, as well as use metagenomes to study gut microbes-mediated pathogen defense mechanisms. Moreover, the effects of food and sex on gut microbial communities in wild birds must be examined. Lactobacilli or other probiotics might be fed artificially during migration. According to the study of Shojadoost et al. (2019), certain lactobacilli species can effectively enhance the antiviral response of chicken macrophages (Shojadoost et al., 2019), while probiotics can protect broilers from potential harmful intestinal microorganisms (Eeckhaut et al., 2016). Therefore, they may also play a role in protecting birds from harmful pathogens. Protecting migratory bird stopover habitats and reducing anthropogenic disturbance, such as from human activities, can improve resources available to wildlife, such as diet, and may alter host gut microbiota and health status (Pekarsky et al., 2021), reducing the likelihood of infection in birds from environmental microbial pathogens.

## Data availability statement

The datasets generated for this study can be accessed from NCBI Sequence Read Archive (SRA), SRR16526618–SRR16526752.

## Ethics statement

All animal sample collection agreements comply with the current laws of China. All studies were approved by the Laboratory Animal Welfare and Ethics Committee of Jilin Agricultural University.

## Author contributions

YJ and RY contributed to the design of the study. RY, JY, and LZ collected the gut content samples. RY, ML, SL, and YJ contributed to the writing of the manuscript. All authors contributed to the article and approved the submitted version.

## Funding

This work was supported by the National Natural Science Foundation of China (grant no. 31872684).

## Conflict of interest

The authors declare that the research was conducted in the absence of any commercial or financial relationships that could be construed as a potential conflict of interest.

## Publisher’s note

All claims expressed in this article are solely those of the authors and do not necessarily represent those of their affiliated organizations, or those of the publisher, the editors and the reviewers. Any product that may be evaluated in this article, or claim that may be made by its manufacturer, is not guaranteed or endorsed by the publisher.
