# Supplementary material for: Effect of sex on the gut microbiota characteristics of passerine migratory birds
Source: Front Microbiol. 2022 Sep 2;13:917373. doi: 10.3389/fmicb.2022.917373 (PMC9478027; doi:10.3389/fmicb.2022.917373)
Supplement: Supplementary file 1 [file Data_Sheet_1.docx]

Supplementary Material

**Supplementary Materials**: amplification system (25μL): 5×reaction buffer 5μL, 5×GC buffer 5μL, dNTP（2.5mM） 2μL, Forward primer （10µM）1μL, Reverse primer （10µM）1μL, DNA Template 2μL, ddH2O 8.75μL, Q5 DNA Polymerase 0.25μL, amplification parameters: initial denaturation 98℃ 2min， denaturation 98℃ 15s， annealing 55℃ 30s， extension 72℃ 30s，30 cycles, final extension 72℃ 5min, 10℃ Hold.

**Ethical Statement:** According to the wildlife protection law of the people of the Republic of China (order of the president of the people of the Republic of China, No. 16, 2018), all animal sample collection agreements comply with the current laws of China. All studies were approved by the Laboratory Animal Welfare and Ethics Committee of Jilin Agricultural University. Fecal samples were collected without harming birds.

**Supplementary Figures**

**Supplementary Figure 1**


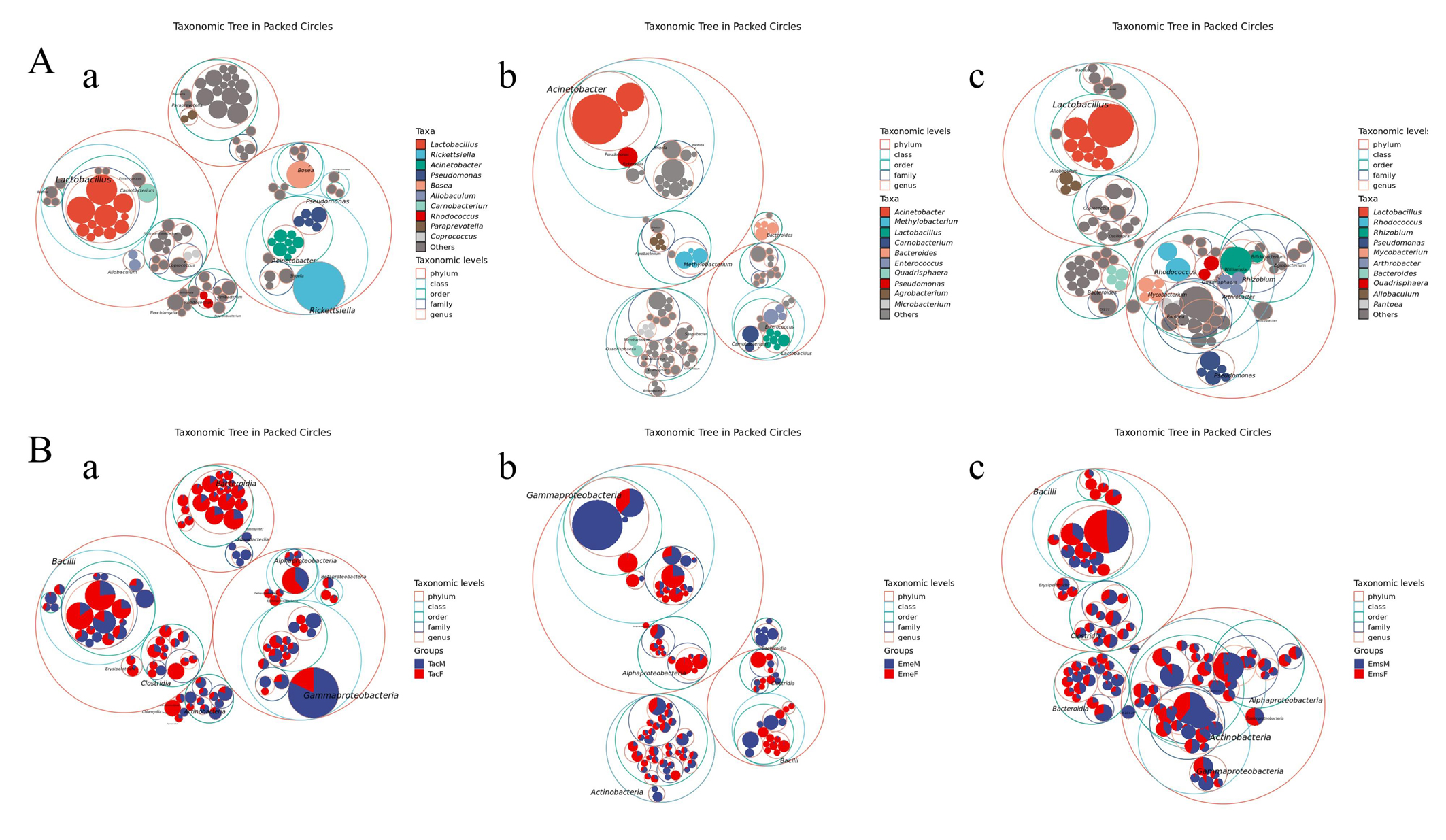


**Supplementary Figure 1**. Taxonomic hierarchy tree diagram. The largest circle represents the phylum level, and the gradual shrinking circles represent classes, orders, families and genera depending on the size reduction. The innermost dots represent the top 100 ASVs in terms of abundance, and their size (area) is proportional to the abundance of that ASV. Therefore, the abundance of the taxonomic unit analogous to the circle can also be identified by the area of the origin within the circle. The larger the area of the sector, the higher the abundance of that taxonomic unit in the corresponding grouping. (Aa) is the composition of the corresponding taxonomic units at the respctive taxonomic level in *T. cyanurus*, (Ab) is the composition of the corresponding taxonomic units at the respective taxonomic level in *E. elegans*, and (Ac) is the composition of the corresponding taxonomic units at the respective taxonomic level in *E. spodocephala*. (Ba) is the composition of the corresponding taxonomic units at the respective taxonomic level in *T. cyanurus* female and male birds, (Bb) is the composition of the corresponding taxonomic units at the respective taxonomic level in *E. elegans* female and male birds, and (Bc) is the composition of the corresponding taxonomic units at the respective taxonomic level in *E. spodocephala* female and male birds. In the figure, Tac=*T. cyanurus*, Eme=*E. elegans*, Ems=*E. spodocephala*; TacM= *T. cyanurus* male, TacF= *T. cyanurus* female; EmeM= *E. elegans* male, EmeF= *E. elegans* female; EmsM= *E. spodocephala* male, EmsF= *E. spodocephala* female.

**Supplementary Figure 2**


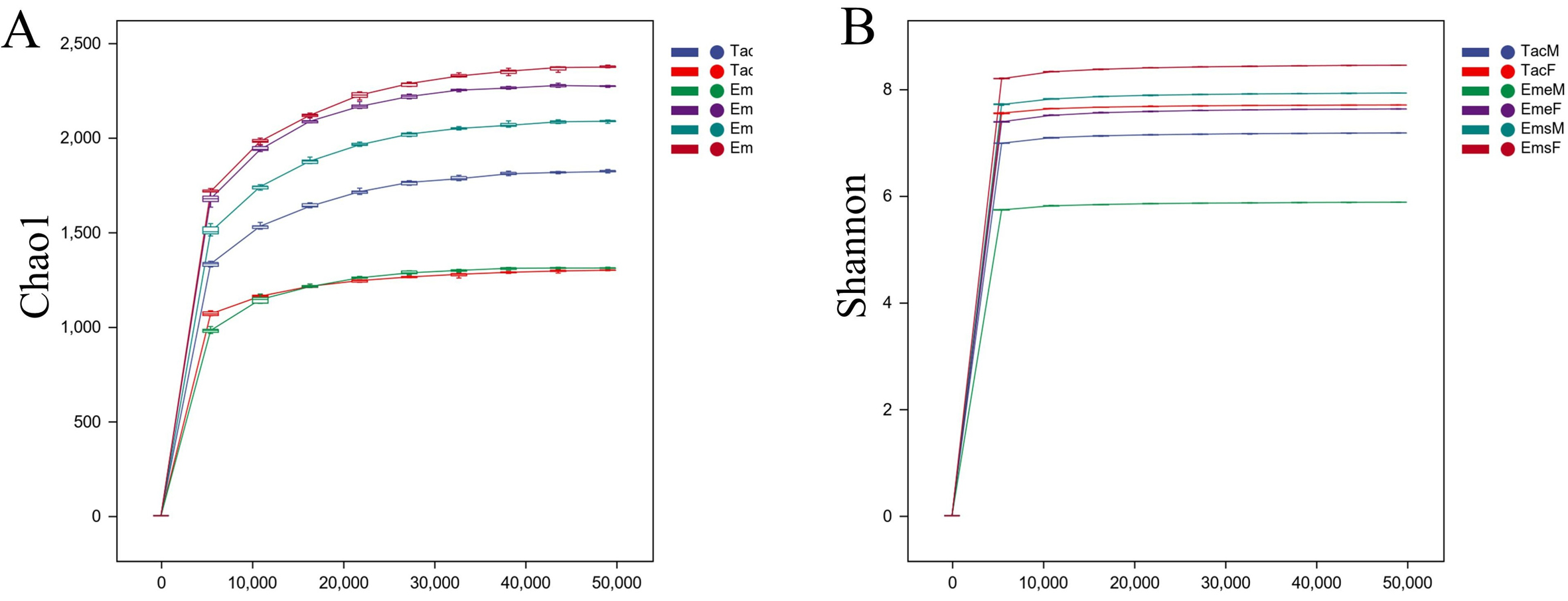


**Supplementary Figure 2.** Sparsity curves. The horizontal coordinate is the drawdown depth and the vertical coordinate is the median value of the alpha diversity index calculated 10 times versus the box line plot. The meaning of the abbreviations in the figure is shown in Supplementary Figure 1.

**Supplementary Figure 3**


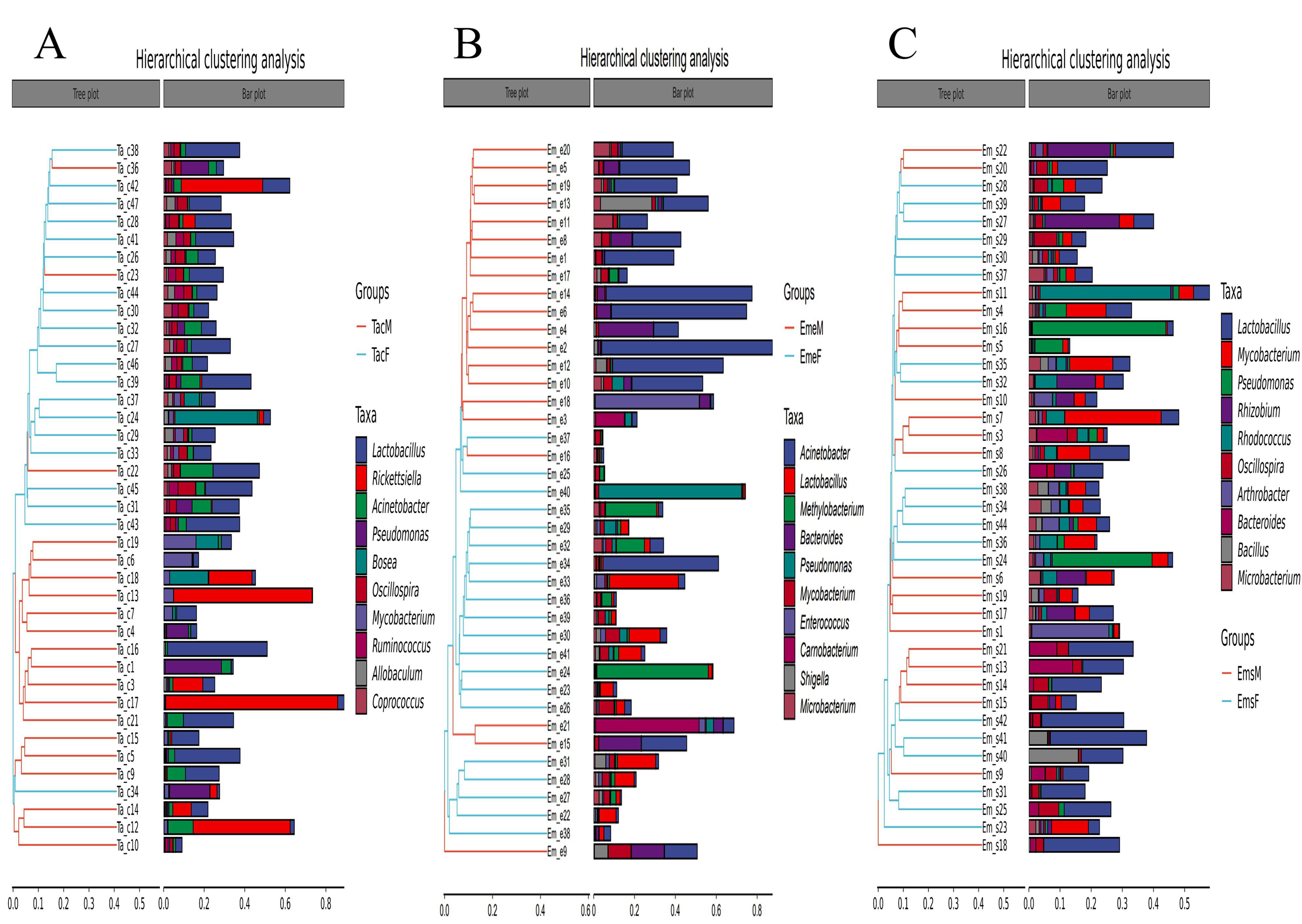


**Supplementary Figure 3**. Average cluster analysis diagrams. The panel on the left is a hierarchical clustering tree, and the panel on the right is a stacked histogram of the top 10 genera in abundance. A is the average cluster diagram of male and female individuals of *T. cyanurus*; B is the average cluster diagram of male and female individuals of *E. elegans*; C is the average cluster diagram of male and female individuals of *E. spodocephala*. The meaning of the abbreviations in the figure is shown in Supplementary Figure 1.

**Supplementary Figure 4**


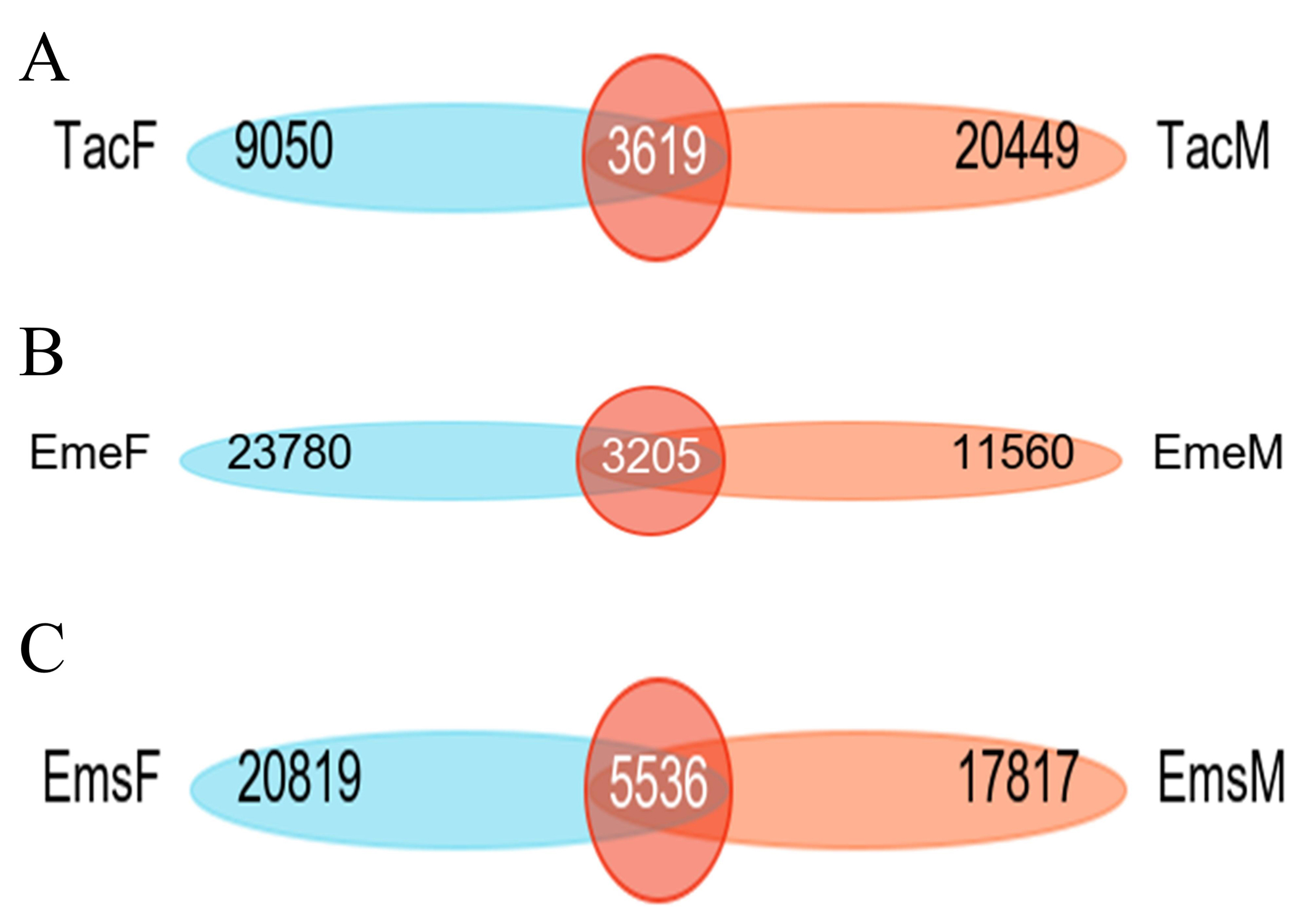


**Supplementary Figure 4.** The Wenn diagram of cluster analysis. In the figure, each color block represents a grouping, the overlapping area between color blocks indicates the ASV shared by the corresponding group, and the number of each block indicates the number of ASV contained in that block. A: Wenn diagram of female and male individuals of *T. cyanurus*, B: Wayne diagram of female and male individuals of *E. elegans*, C: Wayne diagram of female and male individuals of *E. spodocephala*. The meaning of the abbreviations in the figure is shown in Supplementary Figure 1.

**Supplementary Tables**

**Supplementary Table 1**. Differences in alpha diversity. Kruskal-Wallis rank-sum test and Dunn's post hoc test were run for significance. The meaning of the abbreviations in the figure is shown in Supplementary Figure 1.

**Supplementary Table 2**. PERMANOVA test. "Sample size" indicates the sum of the total number of samples compared, "Permutation" indicates the number of permutation tests, "pseudo-F " denotes the value of the statistic F, "p-value" denotes the p-value of the permutation test, and "q-value" is the corrected q-value of the multiple tests. The meaning of the abbreviations in the figure is shown in Supplementary Figure 1.
